# Drinking Water with Saccharin Sodium Alters the Microbiota-Gut-Hypothalamus Axis in Guinea Pig

**DOI:** 10.3390/ani11071875

**Published:** 2021-06-23

**Authors:** Junrong Li, Shanli Zhu, Zengpeng Lv, Hongjian Dai, Zhe Wang, Quanwei Wei, Enayatullah Hamdard, Sheeraz Mustafa, Fangxiong Shi, Yan Fu

**Affiliations:** 1 College of Animal Science, Zhejiang University, Hangzhou 310058, China; lijornsky@163.com; 2 College of Agriculture, Jinhua Polytechnic, Jinhua 321000, China; 2020205012@stu.njau.edu.cn; 3 College of Animal Science and Technology, Nanjing Agricultural University, Nanjing 210095, China; lvzengpeng310@163.com (Z.L.); 2018105021@njau.edu.cn (H.D.); 2018105020@njau.edu.cn (Z.W.); weiquanwei@njau.edu.cn (Q.W.); 2017105117@njau.edu.cn (E.H.); sheerazmustafa786@gmail.com (S.M.)

**Keywords:** saccharin, hypothalamus, gut, microbiota, sweet receptor

## Abstract

**Simple Summary:**

Saccharin sodium (SS) is one of the commonly used artificial sweeteners (AS) in the food industry, but the mechanisms mediating the physiological effects of sweeteners in the gut-brain axis is still unclear. The aim of this study was to explore the regulatory effect of SS on the microbiota-gut-hypothalamus axis on guinea pigs. We found that SS treatment may alter the growth and glucose metabolism of guinea pigs by activating sweet receptor signaling in the gut and growth hormone-releasing peptide (GHRP) hormone secretion. Besides, SS treatment increased the abundance of Firmicutes and Lactobacillasceae-Lactobacillus in the ileum, and subsequently increased levels of lactic acid and short-chain fatty acids (SCFAs). Adding 1.5 mM SS to drinking water alters the growth of guinea pigs by regulating the microbiota-hypothalamus-gut axis. This conclusion has theoretical implications for the comprehensive assessment of the biological effects of appropriate SS in the food industry.

**Abstract:**

The effects of saccharin, as a type of sweetener additive, on the metabolism and development of mammals are still controversial. Our previous research revealed that saccharin sodium (SS) promoted the feed intake and growth of guinea pigs. In this experiment, we used the guinea pig model to study the physiological effect of SS in the microbiota-gut-hypothalamus axis. Adding 1.5 mM SS to drinking water increased the serum level of glucose, followed by the improvement in the morphology and barrier function of the ileal villus, such as SS supplementation which increased the villus height and villus height/crypt depth ratio. Saccharin sodium (SS) treatment activated the sweet receptor signaling in the ileum and altered GHRP hormone secretion. In the hypothalamus of SS and control (CN) group, RNA-seq identified 1370 differently expressed genes (796 upregulated, 574 downregulated), enriching into the taste signaling transduction, and neuroactive ligand–receptor interaction. LEfSe analysis suggested that *Lactobacillaceae-Lactobacillus* was the microbe with significantly increased abundance of ileum microorganisms in the SS-treated group, while *Brevinema-Andersonii* and *Erysipelotrichaceae-Ilebacterium* were the microbes with significantly increased abundance of the control. Furthermore, SS treatment significantly enhanced the functions of chemoheterotrophy and fermentation of ileal microflora compared to the CN group. Accordingly, SS treatment increased levels of lactic acid and short-chain fatty acids (acetic acid, propionic acid and N-valeric acid) in the ileal digesta. In summary, drinking water with 1.5 mM SS activated sweet receptor signaling in the gut and altered GHRP hormone secretion, followed by the taste signaling transduction in the hypothalamus.

## 1. Instruction

Artificial sweeteners (AS) are widely used in food products and soft drinks. Saccharin has good hydrolysis and PH stability, which can be completely absorbed and quickly metabolized [1]. Early intervention studies revealed that the chronic replacement of dietary sugar with AS can reduce energy intake and body weight via reducing calorie intake [2]. It is suggested that AS induces taste receptor activation in the intestine and adaptively regulates the expression of glucose transporters (SGLT-1/GLUT2), which are closely related to the glucose homeostasis [3]. Therefore, many researchers insist that AS ingestion stimulates hunger and consequently increases food intake via activating sweet taste receptors in the small intestine [4,5]. The paradoxical association between the consumption of AS and weight gain mainly focuses on the feed intake, glucose absorption, and gut flora. The importance of nutrient-induced brain-gut signaling in the regulation of animal metabolism and food intake has become increasingly obvious. However, the mechanisms mediating the physiological effects of sweeteners in the gut-brain axis are still unclear.

Gut microflora not only influences intestinal function, but also plays an important role in regulating brain-gut axis signals [6]. An earlier study found that saccharin intake reduced the ratio of anaerobic/aerobic microorganisms in the gut of rats using the first-generation DNA sequencing technology [7]. However, which specific microorganisms are involved is still unclear. Short-chain fatty acids (SCFAs), deriving from bacteria-dependent hydrolysis of fibers, are essential to intestinal epithelial cells, which can modulate their proliferation and differentiation, to influence gut motility and to strengthen the gut barrier functions as well as host metabolism [8]. Furthermore, SCFAs are predicted to have an essential role in microbiota-gut-brain crosstalk [9]. Therefore, exploring the regulatory effects of AS on mammalian growth and metabolism requires comprehensive consideration from the perspective of the microbiota-gut-brain axis. 

Diversity in sweet taste sensation among mammal species occurs also at the level of the gut [10]. Since guinea pig is sensitive to external stimuli, it is a suitable animal model for AS research. What is more, the diet of guinea pig is mainly composed of plant-derived fiber, which makes the content of SCFAs in the intestine extremely vulnerable [11]. Mallett et al. suggested that adding saccharin in the diet could alter the ratio of SCFAs in the cecum of mice and inhibit the hydrolysis of amylase [12]. Therefore, we hypothesized that saccharin sodium might regulate gut development and energy intake through SCFAs. In this experiment, we aim to explore the regulatory effect of saccharin sodium (SS) on the microbiota-gut-hypothalamus axis, which can provide a theoretical basis for studying the effect of AS on adolescent development.

## 2. Materials and Methods

### 2.1. Animal, Diets, and Management

Two feeding trials were conducted for 28 days: a control group which received normal water and a SS group which received water with 5 mM SS solution. The dose of SS was designed the same as in the previous experiments [13]. A total of 12 (4-week-old) female Harley-white guinea pigs (*Cavia porcellus*) with a body weight of 240.7 ± 7.7 g were housed in the Laboratory Animal Research Center of Zhejiang Chinese Medical University (Hangzhou, China) and in accordance with guidelines approved by the National Laboratory Animal Management Regulations (SYXK(ZHE)-2018-0012) and the Institutional Animal Care and Use Committee of Zhejiang Chinese Medical University (IACUC- 20181224–14). Only female guinea pigs were chosen in this study to observe specific effects on female animals. The environmental conditions were set the same as in the previous study [14]. The room temperature was kept at 21 to 23 °C, relative humidity of 30–40%. The treatments were randomly assigned to six replicates each. All guinea pigs were fed the same meal every day in the morning and body weight data were collected every 7 days.

### 2.2. Sample Collection

At the end of the experimental periods, all guinea pigs were euthanized by CO_2_ anesthesia. All guinea pigs were in the luteal phase on the day of sampling to ensure consistent physiological conditions. Blood was collected immediately from the heart and serum was then separated by centrifugation at 1118 g for 10 min at 4 °C. Serum biochemical indices, including TG (triglyceride), ALT (glutamic-pyruvic transaminase), AST (glutamic-oxalacetic transaminase), TC (total cholesterol), CRE (creatinine), ALP (alkaline phosphatase), UA (uric acid), GLU (glucose), CHO (cholesterol), and TP (toll protein) were measured using assay kits (Unicel DXC 800, Breaa, CA, USA). After the hypothalamus and ileum mucosa were collected, the samples were repeatedly washed with DEPC sterilized water and stored in liquid nitrogen and then stored at −80 °C for RNA-Seq and RT-qPCR. The hypothalamic tissue samples were homogenized with a low-temperature high-throughput grinder to extract RNA. The sample collection of ileum followed methods used in previous study [15].

### 2.3. Analysis and Observation of Intestinal Morphological

The ileum segments were fixed in 4% paraformaldehyde for hematoxylin-eosin (HE). The averaged villus height and crypt depth were measured by using ImagePro Plus software version 6.0 (Media Cybernetics, Rockville, MD, USA). For each slide, morphological analyses and observation were performed at magnifications of 50 times.

### 2.4. Lactic Acid and SCFA Detection in the Ileal Digesta

The samples were added into 2 mL of water (1:3 phosphoric acid aqueous solution) and vortex and homogenate samples for 2 min. Then, 1 mL of ether was added in the samples to extract for 10 min, 4000 rpm/min and centrifuge for 20 min under 4 °C, followed by adding 1 mL of ether into solutions to extract and centrifuge for 10 min at 4000 rpm. The two extracts were combined and volatilized to within 1 mL and analyzed for further detection. The concentration of lactic acid (9–5000 U/L) was measured using commercial assay kits (A020-2-2) purchased from Nanjing Jiancheng Bioengineering Institute, Nanjing, China. SCFAs were quantified using a gas chromatography/mass spectrometry analysis (GC/MS, Thermo Fisher ISQTM LT, Thermo Fisher Scientific, Waltham, United State). The SCFAs were separated on a TG WAX capillary column (30 m × 0.25 mm × 0.25 μm) using helium (He) as a carrier gas at a flow rate of 1 mL/min. Column temperature: 100 °C (5 min) −5 °C/min −150 °C (0 min) −30 °C/min −240 °C (30 min). Fatty acid profiles were measured and are expressed in milligrams per gram of total SCFA in the ileal digesta.

### 2.5. RNA Isolation

Hypothalamus and ileal mucosa samples were randomly selected for RNA isolation following methods in previous studies [15]. Total RNA was extracted using Trizol reagent (15596018; Invitrogen LifeTechnologies, Carlsbad, CA, USA) according to the manufacturer’s protocol with the nucleic acid/protein quantitative measuring instrument (Bio-Rad Laboratories, Inc., Hercules, CA, USA). For each individual hypothalamus sample, 3 μg of RNA were used for samples preparation and a NEBNext1 UltraTM RNA Library Prep Kit for Illumina1 (New England Biolabs, Inc., Ipswich, MA, USA) as sequencing libraries was used according to the manufacturer’s protocols. The cDNA was selected at 250–300 bp in length and PCR products were purified by performing AMPure XP system (Beckman Coulter, Beverly, MA, USA). The quality of the library was evaluated on the Agilent Bioanalyzer 2100 system (Agilent Technology Co., Ltd., Santa Clara, CA, China).

### 2.6. RNA-Seq Analysis

The raw data in FASTQ format are processed through internal Perl scripts, and clean data are obtained by removing low-quality data or adapter sequences from the raw data (FASTX-Toolkit). The filtering criteria are adapters, low-quality reads (Qphred <= 20, phred = −log10(e), e(base-calling error rate) = 0.01) at the 3′end, reads with fuzzy N bases, rRNA sequences shorter than 20bps. In order to allow for less than two base mismatches, all double-end data from the two groups were compared with the guinea pig reference genome (Cavia porcellus 3.0, Scaffolds). The reads per kilobase per million (RPKM) were used to calculate gene expression intensity. For this study, if RPKM in one group is >0.3, but is <0.3 in the other group, the gene is only expressed in one of the two groups. The DESeq2 R package (1.16.1) were used to analyze the differential expression of the two groups and the resulting P values needed to be adjusted to control the false discovery rate by using the Benjamini-Hochberg method [16]. The genes were defined as differently expressed genes (DEGs) when the gene expression levels had a *p* value <0.05, fold change >1.5 (increased 1.5 times compared with the control) or 0< fold change <0.67 (decreased 1.5 times compared with the control).

### 2.7. Genes Identification

Gene ontology (GO) and the Kyoto Encyclopedia of Genes and Genomes (KEGG) were used for performing enrichment analysis of the significant overrepresentation of GO items and KEGG pathway categories.

### 2.8. qRT-PCR

Although RNA-Seq is the core technology of gene expression profiling, qRT-PCR is still the preferred technology for verification. qRT-PCR were performed because qRT-PCR validation of microarray involves hybridization to a glass slide and the dynamic range of microarrays has long been known to be constrained compared to qRT-PCR. When RNA-Seq data are based on a small number of biological replicates, performing qRT-PCR on more samples and focusing on some interesting goals is a good way to verify RNA-Seq results and construct studies. Ten DEGs were randomly selected for performing qRT-PCR. Total mRNA was isolated using TRIzol reagent (Invitrogen, Carlsbad, CA, USA) and was reverse-transcribed using the cDNA reverse transcription kit (TaKaRa, Dalian, China) with gDNA Eraser. The one-step real-time RT-PCR (ABI7500; Applied Biosystem, Carlsbad, CA, USA) was performed using SYBR Premix Ex TaqTM and was used to quantify the mRNA expression levels. Supplementary Data 1 show the primer sequence (Primer Express 3.0.1 software). The relative quantitation of a given gene expression was adjusted as the housekeeping gene, β-Actin, using the 2^−^^ΔΔCT^−method [17], and normalized to the control.

### 2.9. 16 S Ribosomal DNA Gene Sequencing

Five samples of ileal digesta were chosen for 16S rDNA gene sequencing analysis and a QIAampDNA Stool Mini Kit (Qiagen Inc., Valencia, CA, USA) was used for DNA extraction, following methods used in previous studies [15]. Barcode-specific primers (16S V4, 515F, GTGCCAGCMGCCGCGGTAA; 806R, GGACTACHVGGGTW TCTAAT) were used to amplify different regions of the 16S rRNA gene. Quantitative Insights Into Microbial Ecology software (v1.7.0, http://qiime.org/ (accessed on 21 December 2020)) was performed to analyze the sequences. The quality control removes the linker sequence contained in the reads, and removed the low-quality reads with a base error rate less than 0.01 (Qphred <= 20). Clean reads refer to the sequence that is finally used for subsequent analysis after filtering the chimera. UPARSE software (v7.0.1001) was used to analyze sequences and they were clustered into operational taxonomic units (OTUs) at a similarity level of 97%. Each operational taxonomy unit (OTU) will have a representative sequence and use the ribosome database item classifier to perform classification. UCHIME was used to identify and remove chimeric sequences. The most abundant sequence in each OTU is designated as representative sequences and ribosomal Database Project (RDP) classifier was used to classify. Ace, Chao, Shannon, and Simpson diversity indices were used to calculate the Alpha calculation index of community richness. Beta diversity was assessed by principal component analysis (PCA) and the significance of separation was analyzed by ANOSIM (R, v2.15.3). The functional potential of bacteria communities was predicted by using Tax4Fun analysis.

### 2.10. Statistical Analyses

Data were presented as the mean ± SD or mean ± SEM (for gene expression). The significant differences were evaluated using test in SPSS 11.0 for Windows. Comparisons of the relative abundances of microbia between the two groups were performed by unpaired Student’s *t*-test. Significant difference was declared when *p* < 0.05. The significance level for difference was *p* < 0.05. The Tax4Fun analysis package was used to compare the sequencing data with the bacterial metagenomics database (SILVA Reference data) for microbial function prediction. The analysis process refers to previous experiment [18]. LEfSe (LDA Effect Size) is used to analyze high-dimensional biomarkers (genes, pathways, and taxa), which can figure out the biological correlation and biomarker with statistical difference between groups.

## 3. Results

### 3.1. Serum Biochemical Indexes

We first assessed the effect of SS on the serum metabolic indices of guinea pigs (Table 1). The SS group resulted in poorer (*p* < 0.05) UA and GLU than the CN group. However, SS supplementation had no significant effects on serum levels of TG, ALT, AST, CRE, ALP, CHO, and TP. The gastrointestinal tract participates in brain–gut interactions through brain–gut peptides, mainly including growth hormone-releasing peptide (GHRP), glucagonlike peptide 1 (GLP-1), cholecystokinin (CCK), peptide-YY (PYY). In the current study, adding SS to drinking water increased the serum level of GHRP (*p* < 0.05) with no significant effects on PPY, GLP1, and CCK.

### 3.2. Morphological Analysis of the Ileum Villus

**Figure 1:**
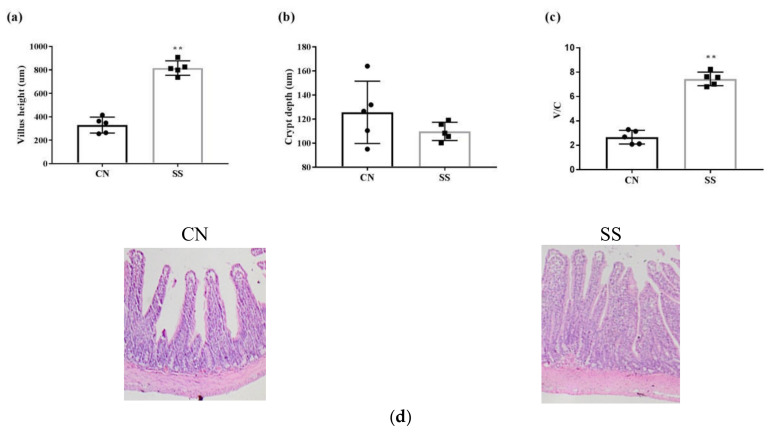
The effects of saccharin sodium on the ileal villus morphology. (**a**) Comparison of ileum villus length between the two groups. (**b**) Comparison of ileum crypt depth between the two groups. (**c**) Comparison of ileum villus height/crypt depth between the two groups. (**d**) Representative photomicrographs of the ileum cross section in guinea pigs. Original magnification is 50×. Values are means, with their standard errors represented by vertical bars, n = 6. ** *p* < 0.01. CN, the control group; SS, the saccharin sodium-treated group.

As is shown in Figure 1, the villus height and villus height/crypt depth ratio were raised by supplementing with SS (*p* < 0.05, Figure 1a,c), along with the decreased crypt depth compared with the CN group (*p* > 0.05, Figure 1b). SS supplementation improved the ileal villus density compared with the CN group based on the photomicrographs of the ileum cross section (Figure 1d).

### 3.3. Gene Expressions of Taste Receptors, Glucose Transporter and Tight Junction in the Ileal Mucosa

**Figure 2:**
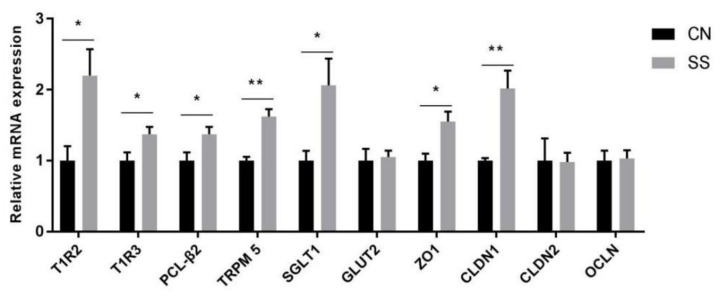
mRNA expressions of genes in the ileal mucosa. Data are presented as mean ± SEM (n = 6) (SPSS 11.0). Abbreviations: T1R2, taste receptor type 1 member 2; T1R3, taste receptor type 1 member 3; PLCβ2, phospholipase Cβ2; TRPM5, transient receptor potential cation channel subfamily M member 5; SGLT1, solute carrier family 5 member 1; GLUT2, solute carrier family 2 member 2; ZO1, tight junction protein 1; CLDN1, claudin 1; CLDN2, claudin 2; OCLN, occludin. CN, the control group; SS, the saccharin sodium-treated group. * *p* < 0.05, ** *p* < 0.01.

Taste receptor type 1 member 2/3 (T1R2/3) is the sweet receptor expressing in the ileum. In the present experiment, SS treatment upregulated the mRNA expressions of T1R2, T1R3, and downstream genes (PLCβ2, phospholipase Cβ2; TRPM5, transient receptor potential cation channel subfamily M member 5; Figure 2, *p* < 0.05). Furthermore, SS treatment exerted significant main effects on upregulating mRNA expressions of solute carrier family 5 member 1 (SGLT1, *p* < 0.05), but with no effects on GLUT2. The tight junction protein is the major indicator to evaluate the intestinal barrier function. The mRNA expressions of tight junction protein 1 (ZO1) and claudin 1 (CLDN1, *p* < 0.05) in the ileal mucosa of SS groups were much higher than those in the CN group. However, there were no significant effects of SS on claudin 2 and occludin.

### 3.4. The Levels of Lactic Acid and SCFAs in the Ileal Digesta

**Figure 3:**
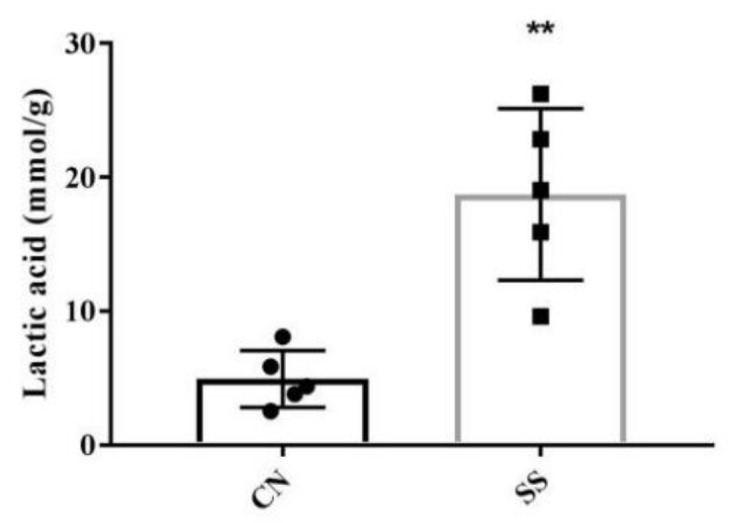
The effects of SS on the lactic acid content in the ileal digesta. Values are means, with their standard deviation represented by vertical bars, n = 5. ** *p* < 0.01. CN, the control group; SS, the saccharin sodium-treated group.

As we can see in Figure 3, adding SS to drinking water significantly increased the level of lactic acid in the ileal digesta (*p* < 0.05). Furthermore, a significant increase of acetic acid, propionic acid, and N-valeric acid was similarly observed in the ileal digesta of SS-treated guinea pigs compared with the CN group (Table 2, *p* < 0.05). Meanwhile, the levels of isobutyric acid (*p* = 0.069) and isovaleric acid (*p* = 0.092) in the ileal digesta of the SS group were higher than that of the CN group. There are no significant effects of SS on the levels of N-butyric acid and N-hexanoic acid in the digesta.

### 3.5. Effects of the Saccharin Sodium Intervention

**Figure 4:**
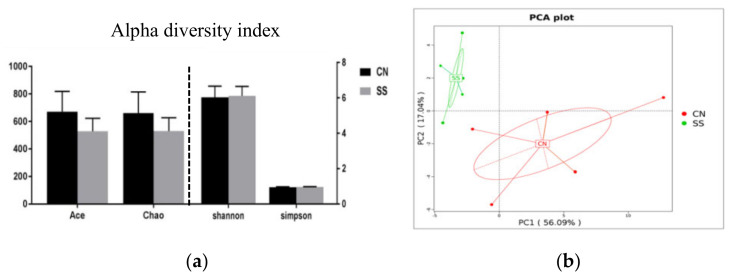
Principle coordinate and diversity analysis of ileal digesta samples, n = 5. (**a**) Effects of saccharin sodium on the diversity of ileal microbiota in guinea pigs. (**b**) Principle coordinate analysis of ileum samples. CN, the control group; SS, the saccharin sodium-treated group.

Sequence data showed that SS supplementation had an impact on the microbial composition of ileal digesta. Each group tested 6 samples, sequence data with >25,000 were considered to be included, which resulted in 5 samples for each group for final analysis. Furthermore, the number of average raw sequence reads detected in each group exceeded 85,411 (Supplementary Data 2). In order to study the species composition of each sample, OTUs (operational taxonomic units) clustering was conducted with 97% identity for the effective tags of all samples, which yielded a total of 6231 OTUs for the entire dataset (Supplementary Data 2). There is no major difference between the diversity indices (Shannon and Simpson) and richness estimators (Ace and Chao) of ileal digesta microbiota in the CN and SS groups (Figure 4a). The PCA analysis indicated that the ileal microbiota composition exhibited significant changes after SS treatment, with a significant separation between the CN and SS groups (*t*-test, *p* < 0.05) (Figure 4b).

**Figure 5:**
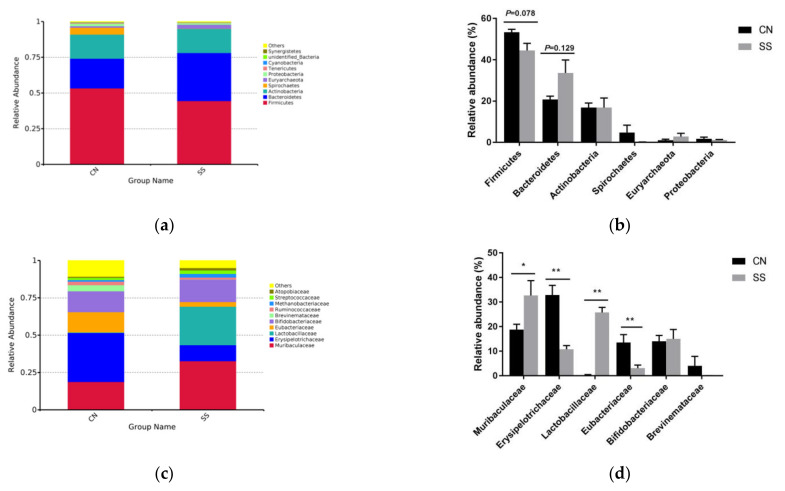

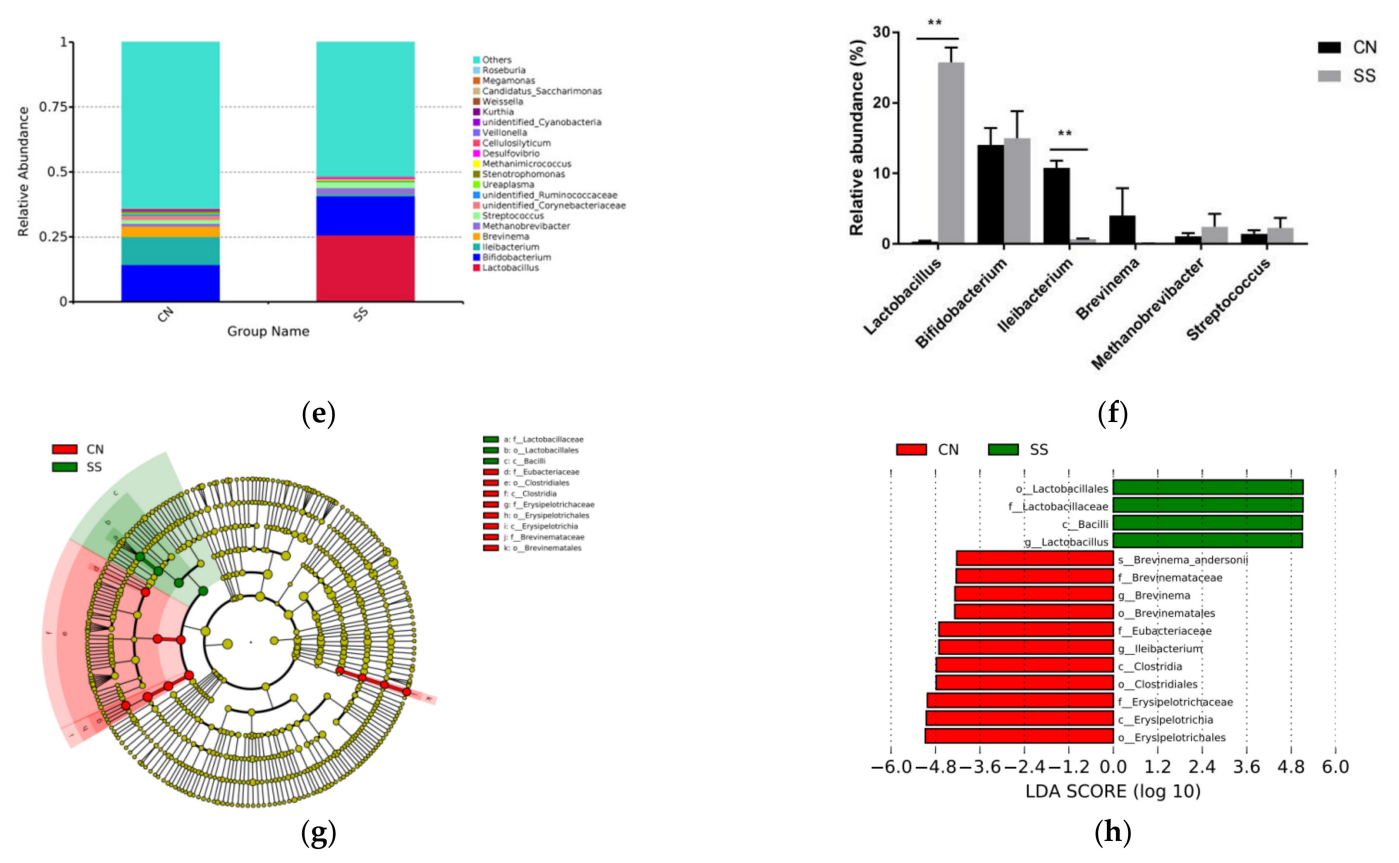
Effects of saccharin sodium on the abundance of ileal microbiota in guinea pigs, n = 5. (**a**,**b**) phylum-level composition; (**c**,**d**) Genus-level composition; (**e**,**f**) Species-level composition. The values are expressed as mean ± SD, n = 5. * *p* < 0.05, ** *p* < 0.01. CN, the control group; SS, the saccharin sodium-treated group. (**g**,**h**) High-dimensional biomarkers (LDA value distribution histogram revealed by LEfSe software. (**b**) Evolutionary branch graph. LDA value distribution histogram indicates species with LDA Score higher than the default setting value (4), which is the biomarker of statistical differences between groups. The length of the histogram (LDA Score) represents the influence on the different species. In the evolution branch diagram, the circles radiating from the inside to the outside represent the classification level from the phylum to genus (or species). Each small circle represents a classification at a different level, the diameter of which is proportional to the relative abundance size. Coloring principle: species with no significant differences are uniformly colored yellow; the color of the species biomarker is the same as the corresponding group. CN, the control group; SS, the saccharin sodium-treated group.

The bacterial composition was evaluated at different taxonomic levels. The dominant bacterial groups were *Firmicutes, Bacteroidetes, Actinobacteria, Spirochaetes, Euryarchaeota,* and *Proteobacteria* at the phylum level. The abundance of Firmicutes tended to decrease in the SS group in comparison with the CN group (Figure 5a,b, *p* = 0.078). In contrast, the abundance of Bacteroidetes in the CN group is lower than that in the SS group (Figure 5a,b, *p* = 0.129). The relative abundance of *Muribaculaceae* (*p* < 0.05) and *Lactobacillaceae* (*p* < 0.01) dramatically increased in the SS group when compared to the CN group (Figure 5c,d) at the family level. In contrast, SS treatment significantly decreased the relative abundance of *Erysipelotrichaceae* and *Eubacteriaceae* (Figure 5c,d, *p* < 0.05). At the genus level, the relative abundance of *Lactobacillus* was significantly increased after SS treatment, along with a decrease in the relative abundance of *Ileibacterium* (Figure 5e,f, *p* < 0.01).

As is shown in Figure 5g,h, Bacilli, Lactobacillales, Lactobacillaceae, and Lactobacillus were the biomarkers in the SS group distinguishing them from the CN group. Meanwhile, Brevinema-andersonii, Brevinemataceae, Brevinema, Brevinematales, Eubacteriacae, ilebacterium, Clostridia, Clostridiales, Erysipelotrichaceae, Erysipelotrichia, and Erysipelotrochales more widely exist in the digesta of the CN group compared with the SS group.

**Figure 6:**
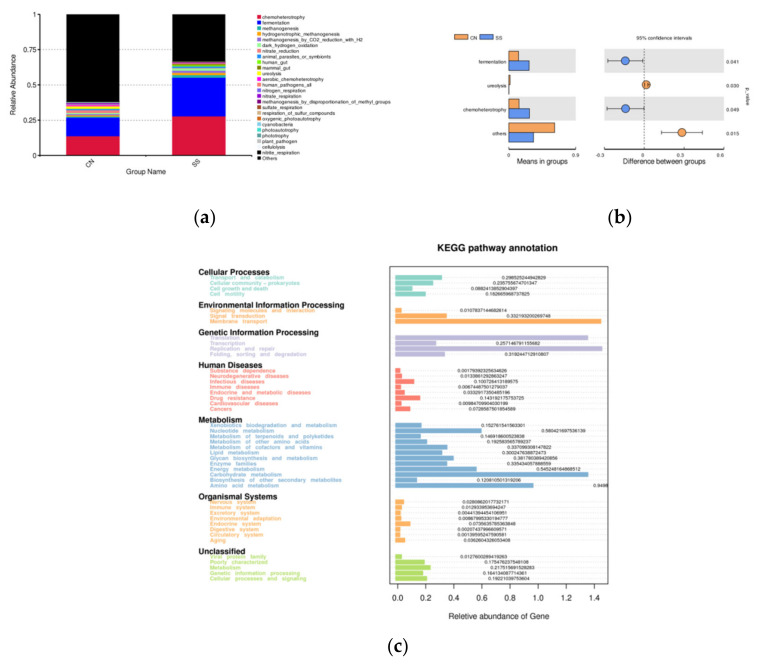
Functional clustering analysis of different OTUs between the SS and CN groups. (**a**) Tax4Fun analysis of the enriched GO functions at the second level. (**b**) The enriched GO functions with significant difference. (**c**) The most important microbial pathways enriched into second-level functional categories using Tax4Fun. CN, the control group; SS, the saccharin sodium-treated group.

Tax4Fun analysis was used to evaluate the changes in the presumptive functions of the ileal microbiota of guinea pigs. Figure 6a shows that the top 25 predicted microbial functions at the second level of the GO enrichment. Chemoheterotrophy, fermentation, methanogenesis, hydrogenotrophic_methanogenesis, methanogenesis_by_CO2_reduction_with_H2, dark_hydrogen_oxidation, nitrate_reduction, animal_parasites_or_symbionts, mammal_gut, ureolysis have top predicted functions with rich Taxa. SS treatment significantly enhanced the functions of chemoheterotrophy and fermentation of ileal microflora compared to the CN group (Figure 6b, *p* < 0.05). The abundance of GO terms related to ureolysis was significantly reduced (Figure 6b, *p* < 0.05). The top abundant microbial pathways enriched into second-level functional categories were about cellular process, environmental information processing, genetic information processing, human diseases, metabolism, organismal system (Figure 6c). Among them, cell growth and death, signaling molecules, carbohydrate and lipid metabolism, nervous system, immune system, and endocrine system were significantly enriched after SS supplementation.

### 3.6. Hypothalamic RNA Sequencing Data and Identification of DEGs in the Hypothalamus between Groups

To study the gene expression profiles in the hypothalamus, we performed RNA-seq analysis of SS-treated guinea pigs and the controls. Note that 52−64 million (M) clean reads from eight RNA-Seq libraries were received to obtain more references to get concise reads with a high proportion of mapped reads ranging from 89.54% to 91.31% after removing the low-quality and adaptor sequences. Most mapped reads were located within an exon (67.01−71.05%), and fewer mapped reads were located within the introns (4.62−6.43%) and intergenic (22.52−26.87%) regions (Supplementary Data 3). These results demonstrated that, compared with the hypothalamus transcriptomes between the SS and CN groups, these 8 libraries had high quality and high coverage of the guinea pig genome.

**Figure 7:**
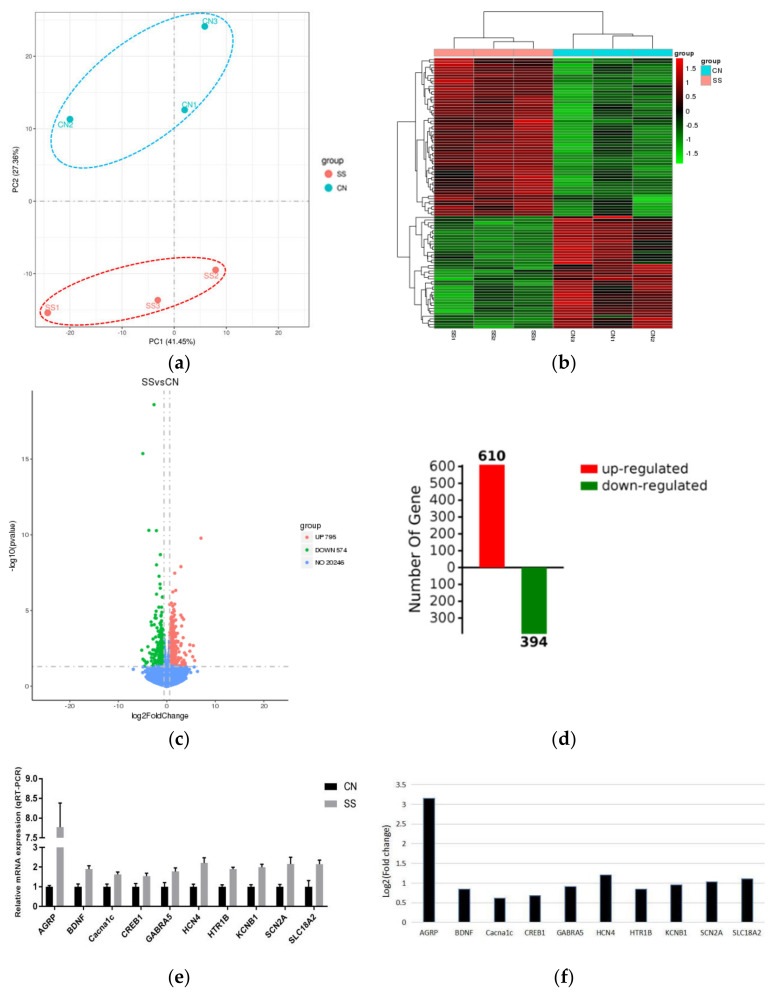
Identification and validation of DEGs in the hypothalamus using RNA-seq and qRT-PCR. (**a**) Principal component analysis (PCA) using gene expressions (FPKM) of all samples. (**b**) The hot map of hierarchical clustering analysis using genes expression values. Red color indicates high gene expression; blue color indicates low gene expression. (**c**) Scatter plot of DEGs (SS vs. CN). Red dots stand for upregulated genes with *p* value < 0.05 and fold change >1.5. Green dots stand for downregulated genes with *p* value < 0.05 and fold change <0.67. (**d**) Omicbean analysis software was used to determine the number of DEGs in the KEGG and GO databases (up-/downregulated) (**e**) qRT-PCR was used to determine relative RNA expressions of random selected genes in the chick hypothalamus. Data are shown as mean ± SEM (n = 6) calculated by ANOVA (SPSS 11.0). (**f**) Cufdiff software (n = 3) was used to determine Log2 (fold change) of gene expression abundance (GE vs. CN) from RNA-seq data. Abbreviations: AGRP, agouti-related neuropeptide; BDNF, brain-derived neurotrophic factor; Cacna1c, calcium voltage-gated channel subunit alpha1 C; CREB1, cAMP responsive element binding protein 1; GABRA5, gamma-aminobutyric acid type A receptor subunit alpha5; HCN4, hyperpolarization activated cyclic nucleotide gated potassium channel 4; HTR1B, 5-hydroxytryptamine receptor 1B; KCNB1, potassium voltage-gated channel subfamily B member 1; SCN2A, sodium voltage-gated channel alpha subunit 2; SLC18A2, solute carrier family 18 member A2; CN, control group; SS, saccharin sodium-treated group.

In the hypothalamus of the SS and CN groups, we identified 1370 DEGs, among which 796 genes were upregulated and 574 genes were downregulated (Figure 7c, |Fold change| >1.5, *p* value < 0.05). All the information of annotated gene and DEGs are shown in Supplementary Data 4. There were 610 upregulated DEGs and 394 downregulated DEGs identified in the KEGG and GO databases using Omicbean analysis software. PCA and hierarchical clustering analysis revealed that 3 SS samples were clustered together and were distinctly different from the clustering of 3 controls (Figure 7a,b). These data demonstrated that SS exhibited unique gene expression profiles in comparison with that of controls.

### 3.7. Function Enrichment Using DEGs of Hypothalamus RNA-Seq

**Figure 8:**
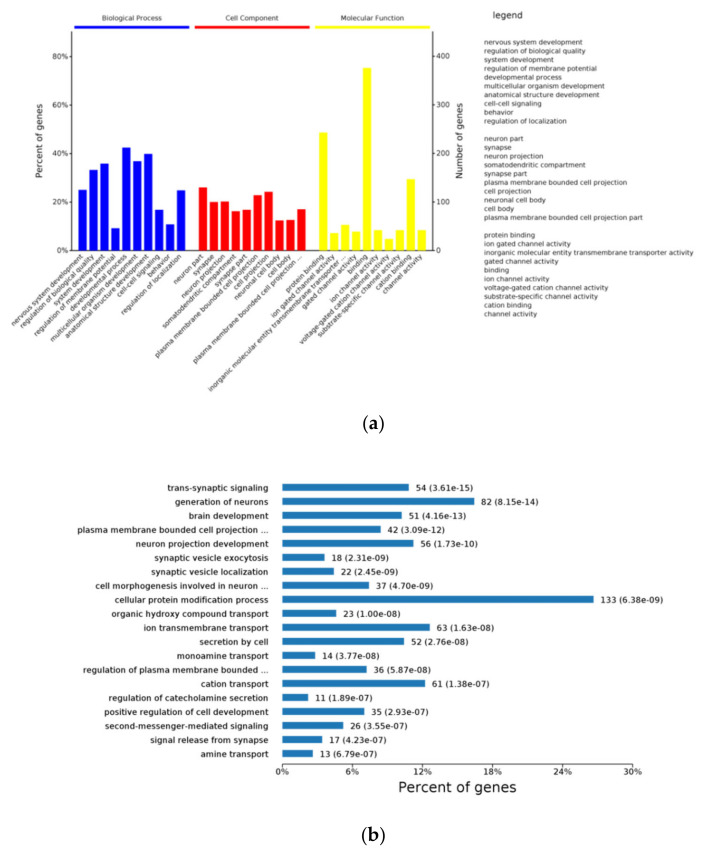

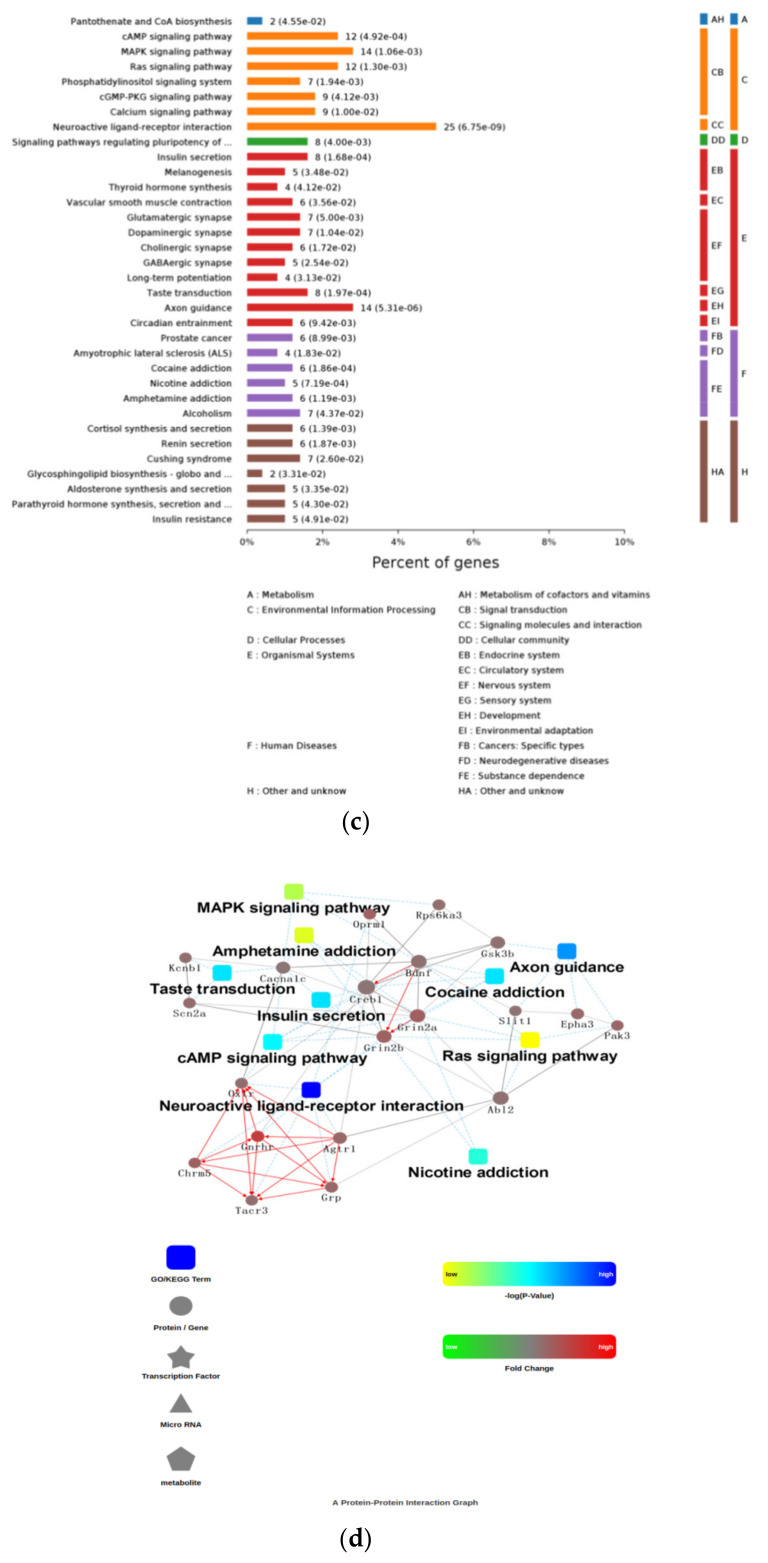
Bioinformatics analysis of RNA-Seq data from the hypothalamus. (**a**) The top 10 enriched items in each main category (biologic process, cell component, and molecular function) of the GO database at all levels using upregulated DEGs between the SS vs. CN groups. (**b**) Level 6 of enriched biological processes with most significant difference. (**c**) Signaling pathway analysis using upregulated DEGs between the SS vs. CN groups. (**d**) Using DEGs of the SS vs. CN groups to analyze protein–protein interaction. Circular nodes stand for genes/proteins; rectangles stand for KEGG pathways or GO biological process terms. The pathways are colored with a gradient from yellow to blue, where yellow demonstrates a smaller *p*-value and blue demonstrates a larger *p* value. GO biological processes are shown in red. Genes/proteins are colored red for upregulation in the fold change analysis.

To further characterize the enriched functions, we performed Gene Ontology (GO) analysis using the upregulated DEGs (Supplementary Data 5). Figure 8a indicates the top 10 enriched terms of biological process, cell component and molecular function at all the GO annotated levels. Importantly, many of these enriched GO terms are closely related to nervous system development (GO:0007399), cell–cell signaling (GO:0007267), neuron part (GO:0097458), synapse (GO:0045202), protein binding (GO:0005515) and cation binding (GO:0043169). At the 6th level (Figure 8b), we can see in detail that DEGs were mostly enriched into trans-synaptic signaling (GO:0099537), generation of neurons (GO:0048699), brain development (GO:0007420), plasma membrane bounded cell projection morphogenesis (GO:0120039), synaptic vesicle exocytosis (GO:0016079), synaptic vesicle localization (GO:0097479), cell morphogenesis involved in neuron differentiation (GO:0048667), cellular protein modification process (GO:0006464), organic hydroxy compound transport (GO:0015850), ion transmembrane transport (GO:0034220), secretion by cell (GO:0032940) monoamine transport (GO:0015844), regulation of plasma membrane bounded cell projection organization (GO:0120035), cation transport (GO:0006812), regulation of catecholamine secretion (GO:0050433), and positive regulation of cell development (GO:0010720). KEGG annotation was used to identify the function of enriched DEGs in the signaling pathways. Figure 8c indicates that DEGs were annotated to 13 KEGG pathways in the hypothalamus (*p* value_adjusted < 0.05). Among them, cAMP signaling pathway, MAPK signaling pathway, Ras signaling pathway, phosphatidylinositol signaling system, and neuroactive ligand–receptor interaction were closely related to signal transduction and signaling molecule interaction. Furthermore, insulin secretion, axon guidance, cocaine addiction, taste transduction, nicotine addiction, phosphatidylinositol signaling system, and amphetamine addiction have the most number of DEGs, indicating that these pathways play important roles in the endocrine system, nervous system, sensory system, and development. In addition, SS treatment enhanced renin secretion, which was related to the hypothalamic thirst by angiotensin stimulation. We validated the expression of 9 genes in these related pathways using real-time qRT-PCR and demonstrated that Cacna1c, Gabra5, Hcn4, Htr1b, Kcnb1, Scn2a, Bdnf, Creb, and Slc18a2 were increased in SS compared with the control.

Protein–protein Interaction Analysis for all upregulated DEGs using Cytoscape bioinformatics was performed (Figure 8d, Supplementary Data 5). The major biologic functions r were significantly related to the categories’ neuroactive ligand–receptor interaction, MAPK signaling pathway, Ras signaling pathway, insulin secretion, axon guidance, cocaine addiction, taste transduction, nicotine addiction, cAMP signaling pathway, and Amphetamine addiction. Among them, Cacnalc, Creb1, Bdnf, GSK3b, Scn2a, Grin2a, Slit1, Abl1, Oxtr, Agtr1, Gnrhr, Chrm5, Tacr3, and Grp are related to the most abundant genes, which can help explain why SS treatment can activate taste-signaling transduction.

## 4. Discussion

Saccharin sodium (SS), as a type of AS, is widely used in human food. Under normal dietary conditions, AS can regulate energy balance through the neuroendocrine system [19]. However, it is unclear whether SS has direct or indirect effects on animal metabolism and growth. The relevant data published by our team revealed that adding 1.5 mM SS to drinking water increased the feed intake and body weight gain of guinea pigs [14]. In the present study, SS treatment significantly increased the serum level of glucose in comparison with the CN group, this increase may affect the energy intake and body weight of guinea pigs. Previous research suggested that the diet with 5% AS caused a significant increase in blood glucose and metabolic disorders in rats [20,21,22]. In the current experiment, SS treatment had no significant effect on the serum levels of TG, CHO, AST, and ALT, indicating that the increase in blood glucose was still within the tolerance range of guinea pigs, and induced no obviously metabolic disorders and abnormal organ function. This difference might be related to the different dose of SS and the tolerance of guinea pigs from other animals.

Sternini et al., have proved that the taste receptor exists in the intestinal endothelial cells and endocrine cells, which can regulate the intestinal cavity and food intake [23]. In the present experiment, SS treatment significantly increased the transcriptional expressions of T1R2, T1R3, and downstream genes (PLC-β2, TRPM5). It is demonstrated that sweet receptor T1R2/T1R3 can increase the glucose absorptive capacity in response to AS through regulating SGLT1 expression [24]. Consistently, drinking water with SS significantly upregulated transcriptional levels of SGLT1 in the ileum, which could explain why blood glucose in the serum increased. The presence of AS can initiate gut-brain signaling via the release of gut hormone and concomitant activation of vagal afferents [25]. Importantly, SS treatment increased the serum level of GHRP. GHRP, as the only type of appetite-stimulating brain gut peptide, is known to promote gastric acid production, gastric motility and emptying. Furthermore, as an endogenous ligand of growth hormone secretagogue type I receptor (GHSR), GHRP can stimulate the pituitary gland to release growth hormone and promote appetite [26]. Therefore, SS treatment might increase the feed intake and body weight through activating sweet receptor signaling and brain–gut hormone secretion.

The hypothalamus is at the core of homeostatic control, which integrates signal input involved in eating behavior [27]. Ventromedial hypothalamic area (VMH) is known as the “satiety center”, expressing orexigenic factors [28]. In the current study, adding SS into the drinking water promoted hypothalamus nervous system development, trans-synaptic signaling, generation of neurons, synaptic vesicle exocytosis, synaptic vesicle localization and sensory system development. Furthermore, SS treatment enhanced expression of genes involved in this processes which was closely associated with signal transduction and signaling molecule interaction. ARC neurons, expressing the neurotransmitter NPY and agouti-related peptide (AGRP), signal to stimulate feeding [29]. Importantly, SS treatment upregulated the expression of AGRP in the hypothalamus in comparison with the control. Furthermore, SS treatment upregulated the transcriptional levels of CREB1, GSK3B, SLC18A2, and Cacna1c, which were enriched into dopaminergic synapse process. Therefore, SS treatment can activate taste signaling transduction, and enhanced neuroactive ligand–receptor interaction through altering the RNA profile in the hypothalamus.

As mentioned above, the optimal adding level of SS had not resulted in obviously metabolic disorders in guinea pigs. Energy intake is not the only one impact factor impacted by SS treatment, there must be other regulatory effectors. The gut microflora is an important participant in the brain–gut axis system, known as the “second brain”, mediating neural signaling exchange and growth development [30]. In the present study, the addition of SS increased the abundance of Firmicutes in the ileum, while reducing the abundance of Bacteroidetes. Bacteroidetes and Firmicutes are the dominant bacteria in the mammal gut. Several studies have suggested that the abundance of Firmicutes in the gut is directly proportional to body weight gain, while Bacteroides is the opposite, which is consistent with the growth phenotype in this experiment [31,32]. Therefore, we concluded that SS might promote the growth of guinea pigs by regulating the gut microflora. What we stress here is that SS treatment increased the family abundance of *Muribaculaceae* and Lactobacillus in the ileum, followed by the increased level of lactic acid. Sufficient evidence revealed that *Lactobacillus* can maintain the micro-ecological balance, inhibit pathogen proliferation in the intestine, and improve immune function [33,34,35]. Bacteria in the family S24-7 (phylum *Bacteroidetes*) are dominant in the gut microbiota of mouse and have been detected in the intestine of other animals [36]. The latest research indicated that S24-7 plays an important role in complex dietary carbohydrate degradation, which is consistent with the elevated blood glucose after SS treatment in this experiment. In contrast, adding SS to drinking water reduced the abundance of *Erysipelotrichaceae* and *Eubacteriaceae*, among which *Ileibacterium* decreased significantly. In particular, the family *Erysipelotrichaceae* is emerging as a group of bacteria that may affect host metabolism and inflammatory diseases, and closely related species have been associated with obesity or protection from weight gain on a high-fat diet [37,38,39]. LEfSe analysis suggested that *Lactobacillasceae-Lactobacillus* was the biomarker of ileum microorganisms in the SS-treated guinea pigs, while *Brevinema-Andersonii* and *Erysipelotrichaceae-Ilebacterium* were the biomarkers of the CN group. This is consistent with the abundance analysis of microflora above. It is further suggested that SS treatment could promote the proliferation of Lactobacillus, and reduce the abundance of *Ilebacterium* in the ileum, which plays an important role in regulating the balance of gut microflora. Gut microbiota will influence liver, brain, and even the metabolism of muscle tissue, finally affecting the whole energy metabolic network in the host (Schroeder and Bäckhed 2016). In the present research, KEGG pathway analysis suggested that SS treatment influenced cell growth and death, signaling molecules, carbohydrate and lipid metabolism, the nervous system, immune system, and endocrine system. In addition, the SS group exhibited significantly less abundance of GO terms related to ureolysis. This was consistent with the decrease of serum urea in SS-treated guinea pigs. Therefore, gut microflora plays an essential role in the regulation of growth and metabolism after SS treatment.

Gut microbiota is an essential barrier for the host and plays vital roles in the digestion and fermentation of carbohydrates, intestinal villi development, and immune response [40]. The metabolites produced by the bacterial fermentation of diets, namely SCFAs, play an essential role in linking host nutrients to intestinal homoeostasis maintenance. Furthermore, due to their neuroactive properties and their influence on other gut-brain signaling pathways, including the immune and endocrine systems, SCFAs may directly or indirectly participate in communication along the microbiota-gut-brain axis [9]. Mallett et al. suggested that the diet with 5% or 7.5% saccharin increased digesta content in rats, followed by the increased level of lactic acid [11]. In the current experiment, adding SS to drinking water significantly increased the lactic acid level in the digesta, which is consistent with the increase of *Lactobacillus* in the ileum and fermentation process enriched by GO analysis. As we all know, *Lactobacillus* produces organic acids, enzymes and acidophilins by fermentation process to improve nutrient potency and growth performance [41,42]. Further detection revealed that SS treatment increased the content of acetic acid, propionic acid, N-valeric acid, N-butyric acid, and N-hexanoic acid in the ileal digesta. Acetate is a fermentation product for most gut bacteria, while butyrate and propionate are produced by more specific bacterial species. Propionate is mainly involved in the process of gluconeogenesis [43], while acetate and butyrate are mainly involved in lipid biosynthesis [44]. It is reported that the amounts of acetate and propionate correlate positively with *Bacteroidetes* within *Firmicutes* [45,46]. This is consistent with the change of ileal microflora in our experiment. Through glycoside hydrolases, polysaccharide lyases, and carbohydrate esterases, gut-associated bacterial communities associated with the intestine can decompose and ferment complex carbohydrates into SCFAs [46]. Butyrate is produced from acetate, lactate, amino acids, and various carbohydrates via glycolysis. In the present study, the change of butyrate in the digesta is consistent with that of lactic acid and acetic acid, which are associated with the enriched process of complex carbohydrate degradation. Therefore, SS treatment could promote the production of lactic acid and SCFAs by regulating gut microflora. Besides, SCFAs act as specific G protein-coupled receptor (GPR) signaling molecules to regulate glucose metabolism [47]. This also explains the consistency of changes in ileal SCFA content and serum biochemical indexes after SS treatment. Furthermore, previous study indicated that SCFAs can decrease pH of the gut and restrain the colonization and proliferation of some pathogens [48]. In this current study, adding SS to drinking water significantly inhibited the abundance of pathogenic bacteria in the ileum. Therefore, adding 1.5 mM SS to drinking water is beneficial to improve the intestinal health of guinea pigs.

Previous studies demonstrated that SCFAs play an essential role in improving the morphology and function of epithelial cells [8]. For example, butyrate, as the main energy source of intestinal cells, is metabolized by hindgut cells. Accordingly, we found that SS supplementation increased the villus height and villus height/crypt depth ratio. The increase in the intestinal villi height can reflect the increased number of mature villi cells and absorptive capacity. The high value of villus height/crypt depth ratio indicates that the intestine has strong digestive and absorption capacity. Previous studies suggested that SS treatment could produce more SCFAs by regulating gut microflora and improve intestinal villi morphology. Similarly, the dietary fiber is fermented, resulting in SCFAs, which promote proliferation of the mucosal epithelium and villus height [49]. Previous studies indicated that SCFAs (acetate, propionate, and butyrate) produced by the microbiota as final metabolites can improve gut barrier function [50]. Supplementing butyrate to diet can inhibit the disruption of the intestinal epithelial barrier induced by high-fat diet by upregulating CLDN1 gene expression [51]. Similarly, SS treatment significantly increased the mRNA expressions of zonula occludes protein, including ZO1 and CLDN1, which can enhance the barrier function of intestinal epithelial cells. Therefore, SS supplementation might enhance intestinal barrier function via regulating SCFAs.

## 5. Conclusions

In conclusion, the microbiota-gut-hypothalamus axis plays an important role in the regulatory effect of SS on the growth and glucose metabolism of guinea pigs. SS treatment activated sweet receptor signaling in the gut and altered GHRP hormone secretion, followed by the taste signaling transduction in the hypothalamus. Importantly, SS treatment increased the abundance of Firmicutes and Lactobacillasceae-Lactobacillus in the ileum, followed by the increased levels of lactic acid and SCFAs. Therefore, adding 1.5 mM SS to drinking water is beneficial to promote the growth of guinea pigs through regulating the microbiota–hypothalamus–gut axis. This finding is of theoretical significance for comprehensively evaluating the biological effects of appropriate levels of saccharin in food and drinks.

## Figures and Tables

**Table 1:** The effects of saccharin sodium on the serum biochemical indexes and brain–gut peptide.

Indexes	CN	SS	*p*-Value
TG (mmol/L)	0.75 ± 0.09	0.83 ± 0.07	0.488
ALT (U/L)	71.3 ± 7.0	86.9 ± 8.2	0.168
AST (U/L)	105 ± 11	114 ± 14	0.614
CRE (μmol/L)	17.1 ± 3.9	11.6 ± 3.2	0.282
ALP (U/L)	262 ± 26	295 ± 27	0.392
UA (μmol/L) *	86.4 ± 14.1	121.3 ± 8.7	0.05
GLU (mmol/L) *	10.2 ± 0.3	12.4 ± 0.4	0.001
CHO (mmol/L)	0.83 ± 0.04	0.80 ± 0.04	0.646
TP (mmol/L)	48.1 ± 2.2	49.5 ± 3.4	0.73
CCK (pg/mL)	38.8 ± 5.2	43.2 ± 5.3	0.136
PPY(pg/mL)	35.8 ± 2.9	34.0 ± 5.6	0.456
GHRP(pg/mL) *	54.8 ± 3.5	60.8 ± 4.3	0.014
GLP1(pg/mL)	4.50 ± 0.72	4.15 ± 0.75	0.347

The results are expressed as the mean ± SD, the significance level for difference is *p* < 0.05 (*t*-test, n = 6). TG, triglyceride; ALT, glutamic-pyruvic transaminase; AST, glutamic-oxalacetic transaminase; TC, total cholesterol; CRE, creatinine; ALP, alkaline phosphatase; UA, uric acid; GLU, glucose; CHO, cholesterol; TP, toll protein. * indicates significance difference.

**Table 2:** The effects of saccharin sodium on short-chain fatty acids in the ileal digesta.

Groups	Acetic Acid * (mg/kg)	Propionic Acid * (mg/kg)	Isobutyric Acid (mg/kg)	N-Butyric Acid (mg/kg)	Isovaleric Acid (mg/kg)	N-Valeric Acid * (mg/kg)	N-Hexanoic Acid (mg/kg)
CN	800 ± 31	484 ± 23	26.2 ± 0.7	587 ± 148	18.2 ± 1.5	47.8 ± 1.3	2.60 ± 0.28
SS	1021 ± 71	625 ± 28	33.9 ± 7.0	695 ± 77	24.3 ± 6.9	60.8 ± 4.3	2.77 ± 0.59
*p*-value	<0.001	<0.001	0.069	0.183	0.092	<0.001	0.589

Unit: mg/kg. Values were expressed as the mean ± SD, and differences were considered significant when *p* < 0.05 as analyzed by *t*-test (n = 5). CN, the control group; SS, the saccharin sodium-treated group. * indicates significance difference.

## Data Availability

The data that support the findings of this study are available from the corresponding author upon reasonable request.

## Supplementary Materials

The following are available online at https://www.mdpi.com/article/10.3390/ani11071875/s1, Supplementary Data 1: Primers used for quantitative real-time PCR analysis. Supplementary Data 2: Quality control and Tags statistics of 16s rDNA analysis. Supplementary Data 3: Reads statistics of RNA-Seq data. Supplementary Data 4: Gene expression abundance and difference analysis of hypothalamus samples. Supplementary Data 5: Function enrichment using DEGs between the SS and CN groups.

## References

[1] DuBois G.E., Prakash I., Doyle M.P., Klaenhammer T.R. (2012). Non-Caloric Sweeteners, Sweetness Modulators, and Sweetener Enhancers. Annual Review of Food Science and Technology.

[2] Fernstrom J.D., Doyle M.P., Klaenhammer T.R. (2015). Non-nutritive sweeteners and obesity. Annual Review of Food Science and Technology.

[3] Dyer J., Wood I.S., Palejwala A., Ellis A., Shirazi-Beechey S. (2002). Expression of monosaccharide transporters in intestine of diabetic humans. Am. J. Physiol. Liver Physiol..

[4] Fowler S.P., Williams K., Resendez R.G., Hunt K.J., Hazuda H.P., Stern M.P. (2008). Fueling the Obesity Epidemic? Artificially Sweetened Beverage Use and Long-term Weight Gain. Obesity.

[5] Egan J.M., Margolskee R.F. (2008). Taste Cells of the Gut and Gastrointestinal Chemosensation. Mol. Interv..

[6] Cryan J.F., O’Riordan K.J., Cowan C.S.M., Sandhu K.V., Bastiaanssen T.F.S., Boehme M., Codagnone M.G., Cussotto S., Fulling C., Golubeva A.V. (2019). The Microbiota-Gut-Brain Axis. Physiol. Rev..

[7] Anderson R., Kirkland J. (1980). The effect of sodium saccharin in the diet on caecal microflora. Food Cosmet. Toxicol..

[8] Jha R., Fouhse J.M., Tiwari U.P., Li L., Willing B.P. (2019). Dietary Fiber and Intestinal Health of Monogastric Animals. Front. Vet. Sci..

[9] Dalile B., Van Oudenhove L., Vervliet B., Verbeke K. (2019). The role of short-chain fatty acids in microbiota-gut-brain communication. Nat. Rev. Gastroenterol. Hepatol..

[10] Tan H.-E., Sisti A.C., Jin H., Vignovich M., Villavicencio M., Tsang K.S., Goffer Y., Zuker C.S. (2020). The gut-brain axis mediates sugar preference. Nat. Cell Biol..

[11] Patten G.S., Bird A.R., Topping D.L., Abeywardena M.Y. (2004). Effects of convenience rice congee supplemented diets on guinea pig whole animal and gut growth, caecal digesta SCFA and in vitro ileal contractility. Asia Pac. J. Clin. Nutr..

[12] Mallett A., Rowland I., Bearne C. (1985). Modification of rat caecal microbial biotransformation activities by dietary saccharin. Toxicology.

[13] Jiang J., Qi L., Wei Q., Shi F. (2018). Effects of daily exposure to saccharin sodium and rebaudioside A on the ovarian cycle and steroidogenesis in rats. Reprod. Toxicol..

[14] Li J., Shen T., Shi F., Fu Y. (2020). Influences of non-nutritive sweeteners on ovarian and uterine expression of T1R2 and T1R3 in peripubertal female guinea pigs. Anim. Sci. J..

[15] Lv Z., Hu C., Jiang J., Jin S., Wei Q., Wei X., Yu D., Shi F. (2019). Effects of High-Dose Genistein on the Hypothalamic RNA Profile and Intestinal Health of Female Chicks. J. Agric. Food Chem..

[16] Benjamini Y., Hochberg Y. (1995). Controlling the false discovery rate: A practical and powerful approach to multiple testing. J. R. Stat. Soc..

[17] Yan H., Zhang Y., Xiong Y., Chen Q., Liang H., Niu M., Guo B., Li M., Zhang X., Li Y. (2018). Selection and Validation of Novel RT-qPCR Reference Genes under Hormonal Stimuli and in Different Tissues of Santalum album. Sci. Rep..

[18] Aßhauer K.P., Wemheuer B., Daniel R., Meinicke P. (2015). Tax4Fun: Predicting functional profiles from metagenomic 16S rRNA data. Bioinformatics.

[19] Gassmann B. (2005). Sweeteners and metabolic syndrome. Ernahr. Umsch..

[20] Palmnäs M.S.A., Cowan T.E., Bomhof M.R., Su J., Reimer R.A., Vogel H.J., Hittel D.S., Shearer J. (2014). Low-Dose Aspartame Consumption Differentially Affects Gut Microbiota-Host Metabolic Interactions in the Diet-Induced Obese Rat. PLoS ONE.

[21] Swithers S.E., Laboy A.F., Clark K., Cooper S., Davidson T. (2012). Experience with the high-intensity sweetener saccharin impairs glucose homeostasis and GLP-1 release in rats. Behav. Brain Res..

[22] Collison K.S., Makhoul N.J., Zaidi M., Saleh S.M., Andres B., Inglis A., Al-Rabiah R., Al-Mohanna F.A. (2012). Gender Dimorphism in Aspartame-Induced Impairment of Spatial Cognition and Insulin Sensitivity. PLoS ONE.

[23] Sternini C., Anselmi L., Rozengurt E. (2008). Enteroendocrine cells: A site of ‘taste’ in gastrointestinal chemosensing. Curr. Opin. Endocrinol. Diabetes Obes..

[24] Margolskee R.F., Dyer J., Kokrashvili Z., Salmon K.S.H., Ilegems E., Daly K., Maillet E., Ninomiya Y., Mosinger B., Shirazi-Beechey S.P. (2007). T1R3 and gustducin in gut sense sugars to regulate expression of Na+-glucose cotransporter 1. Proc. Natl. Acad. Sci. USA.

[25] Jiang P., Cui M., Zhao B., Liu Z., Snyder L.A., Benard L.M.J., Osman R., Margolskee R.F., Max M. (2005). Lactisole Interacts with the Transmembrane Domains of Human T1R3 to Inhibit Sweet Taste. J. Biol. Chem..

[26] Al Massadi O., Nogueiras R., Dieguez C., Girault J.-A. (2019). Ghrelin and food reward. Neuropharmacology.

[27] Berthoud H.-R. (2008). Vagal and hormonal gut-brain communication: From satiation to satisfaction. Neurogastroenterol. Motil..

[28] Konturek S.J., Konturek J.W., Pawlik T., Brzozowski T. (2004). Brain-gut axis and its role in the control of food intake. J. Physiol. Pharmacol. Off. J. Pol. Physiol. Soc..

[29] Han Y., Xia G., Wu Q. (2018). Functional Interrogation of the AgRP Neural Circuits in Control of Appetite, Body Weight, and Behaviors. Advances in Experimental Medicine and Biology.

[30] Liu S., Marcelin G., Blouet C., Jeong J.H., Jo Y.-H., Schwartz G.J., Chua S. (2018). A gut-brain axis regulating glucose metabolism mediated by bile acids and competitive fibroblast growth factor actions at the hypothalamus. Mol. Metab..

[31] Turnbaugh P.J., Ley R.E., Mahowald M.A., Magrini V., Mardis E.R., Gordon J.I. (2006). An obesity-associated gut microbiome with increased capacity for energy harvest. Nat. Cell Biol..

[32] Backhed F., Ding H., Wang T., Hooper L.V., Koh G.Y., Nagy A., Semenkovich C.F., Gordon J.I. (2004). The gut microbiota as an environmental factor that regulates fat storage. Proc. Natl. Acad. Sci. USA.

[33] Wang M., Wu H., Lu L., Jiang L., Yu Q. (2020). Lactobacillus reuteri Promotes Intestinal Development and Regulates Mucosal Immune Function in Newborn Piglets. Front. Veter Sci..

[34] Nii T., Kakuya H., Isobe N., Yoshimura Y. (2020). Lactobacillus reuteri Enhances the Mucosal Barrier Function against Heat-killed Salmonella Typhimurium in the Intestine of Broiler Chicks. J. Poult. Sci..

[35] Li S., Qi C., Zhu H., Yu R., Xie C., Peng Y., Yin S.-W., Fan J., Zhao S., Sun J. (2019). Lactobacillus reuteri improves gut barrier function and affects diurnal variation of the gut microbiota in mice fed a high-fat diet. Food Funct..

[36] Lagkouvardos I., Lesker T.R., Hitch T.C.A., Gálvez E.J.C., Smit N., Neuhaus K., Wang J., Baines J.F., Abt B., Stecher B. (2019). Sequence and cultivation study of Muribaculaceae reveals novel species, host preference, and functional potential of this yet undescribed family. Microbiome.

[37] Cox L.M., Sohn J., Tyrrell K.L., Citron D.M., Lawson P.A., Patel N.B., Iizumi T., Perez-Perez G.I., Goldstein E.J.C., Blaser M.J. (2017). Corrigendum: Description of two novel members of the family Erysipelotrichaceae: Ileibacterium valens gen. nov., sp. nov. and Dubosiella newyorkensis, gen. nov., sp. nov., from the murine intestine, and emendation to the description of Faecalibacterium rodentium. Int. J. Syst. Evol. Microbiol..

[38] Woting A., Pfeiffer N., Loh G., Klaus S., Blaut M. (2014). Clostridium ramosum Promotes High-Fat Diet-Induced Obesity in Gnotobiotic Mouse Models. MBio.

[39] Cox L.M., Yamanishi S., Sohn J., Alekseyenko A., Leung J., Cho I., Kim S.G., Li H., Gao Z., Mahana D. (2014). Altering the Intestinal Microbiota during a Critical Developmental Window Has Lasting Metabolic Consequences. Cell.

[40] Schroeder B., Bäckhed B.O.S.F. (2016). Signals from the gut microbiota to distant organs in physiology and disease. Nat. Med..

[41] Grunewald K.K., Mitchell L.K. (1983). Growth of Mice Fed Milk Fermented with Lactobacillus acidophilus1. J. Food Prot..

[42] Schwarzer M., Makki K., Storelli G., Machuca-Gayet I., Srutkova D., Hermanova P., Martino M.E., Balmand S., Hudcovic T., Heddi A. (2016). Lactobacillus plantarum strain maintains growth of infant mice during chronic undernutrition. Science.

[43] Zhang Q., Koser S.L., Donkin S.S. (2016). Propionate induces mRNA expression of gluconeogenic genes in bovine calf hepatocytes. J. Dairy Sci..

[44] Rios-Covian D., Ruas-Madiedo P., Margolles A., Gueimonde M., De los Reyes-Gavilán C.G., Salazar N. (2016). Intestinal Short Chain Fatty Acids and their Link with Diet and Human Health. Front. Microbiol..

[45] Shah H.N., Collins M.D. (1989). Proposal To Restrict the Genus Bacteroides (Castellani and Chalmers) to Bacteroides fragilis and Closely Related Species. Int. J. Syst. Bacteriol..

[46] Flint H.J., Scott K.P., Duncan S.H., Louis P., Forano E. (2012). Microbial degradation of complex carbohydrates in the gut. Gut Microbes.

[47] Besten G.D., Lange K., Havinga R., Van Dijk T.H., Gerding A., Van Eunen K., Müller M., Groen A.K., Hooiveld G., Bakker B.M. (2013). Gut-derived short-chain fatty acids are vividly assimilated into host carbohydrates and lipids. Am. J. Physiol. Liver Physiol..

[48] Shibata N., Kunisawa J., Kiyono H. (2017). Dietary and Microbial Metabolites in the Regulation of Host Immunity. Front. Microbiol..

[49] Knudsen K.E.B., Hedemann M.S., Lærke H.N. (2012). The role of carbohydrates in intestinal health of pigs. Anim. Feed Sci. Technol..

[50] Keeney K.M., Finlay B.B. (2011). Enteric pathogen exploitation of the microbiota-generated nutrient environment of the gut. Curr. Opin. Microbiol..

[51] Matheus V., Monteiro L., Oliveira R., Maschio D., Collares-Buzato C. (2017). Butyrate reduces high-fat diet-induced metabolic alterations, hepatic steatosis and pancreatic beta cell and intestinal barrier dysfunctions in prediabetic mice. Exp. Biol. Med..

