# Supplementary material for: Drinking Water with Saccharin Sodium Alters the Microbiota-Gut-Hypothalamus Axis in Guinea Pig
Source: Animals (Basel). 2021 Jun 23;11(7):1875. doi: 10.3390/ani11071875 (PMC8300211; doi:10.3390/ani11071875)
Supplement: Supplementary file 1 [file animals-11-01875-s001.zip › Supplemental file/Supplementary data 1.pdf]

Primers used for quantitative real-time PCR analysis

ID		Primer sequence (5′→ 3′)	prodSize
	FORWARD	CAACCAGGTGCCAGTATCTCAGTG	
TAS1R3	REVERSE	GTCATAGCAGCAGGAGTGGAAGC	88
	FORWARD	CCTGGCACACGGAAGACAACAC	
TAS1R2	REVERSE	CATCTGCGGTCTGATTGAGGAAGG	150
	FORWARD	GTTCAACGGACAGAGCGGCTAC	
PLC-β2	REVERSE	ACCACATCAATGCGATCCACAGAG	96
	FORWARD	AGCAGAAGCCTCATCTCCAGTCC	
ZO-1	REVERSE	TTGTGGTGAGTGAGAGGAGGAAGG	145
	FORWARD	GGCAAAGTGAACAGCAAACAGTCC	
OCLN	REVERSE	GTCGTCCACAGAGGAGGTCAGAG	105
	FORWARD	TGACACCCAGGCTGCCCAAG	
CLDN2	REVERSE	ACTCCACCCACTACTGCCACTC	144
	FORWARD	GCCTGATCGCCATCTTCGTGTC	
CLDN1	REVERSE	GCCATTCGCATCTTCTGGACCTC	85
	FORWARD	CTGGGTATGGAATCCTGTGGCATC	
Actin beta	REVERSE	CAGCACTGTGTTGGCATAGAGGTC	99
	FORWARD	TCTGGAGGAGGTGATGATGGATGC	
TRPM5	REVERSE	AGCCGCCCGTAAGTCAGGAAG	108
	FORWARD	CTTCCTGGTGGTGATGGCTGTTG	
SGLT1	REVERSE	CCTGCCAAGAAGAAGCCTCCAAC	81
	FORWARD	CACTGTCTTCACTGCGGTCCTG	
GLUT2	REVERSE	TTGTTGGTAGCCTTTCGGTCATCC	138
	FORWARD	AGTCACCCTCCCACGAGAAGTTG	
Cacna1c	REVERSE	GGCAGTGTTGTTGGCGTTGTTG	101
Gabra5	FORWARD	TGTGCTATGCGTTCGTGTTCTCTG	80

	REVERSE	ATCCCAAGCCCAGCCTCTCTTC	
	FORWARD	TCAACGAGGTGCTGGAGGAGTAC	
Hcn4	REVERSE	TGGACTTTGTGGAGGAGGATGGAG	112
	FORWARD	GAGGTGCTGGACTGCTTAGTGAAC	
Htr1b	REVERSE	CATAGAGGGCGATGAGGAGTAGGG	103
	FORWARD	GTTCATCGTCCTCTCCACCATTGC	
Kcnb1	REVERSE	CTGCGGGTTGTCTGTGTTCTGG	94
	FORWARD	GGAGGAGGCAGAACAGAAAGAAGC	
Scn2a	REVERSE	TCCGATCCCACCAGCACCAC	136
	FORWARD	AACTCCCAGTGCCGAACTACCC	
Bdnf	REVERSE	CCTTATGAACCGCCAGCCGATTC	84
	FORWARD	CACCTGCCATCACCACTGTAACG	
Creb	REVERSE	GAATTGCTCCTCCCTGGGTAATGG	90
	FORWARD	TGCTGGACAACATGCTGCTCAC	
Slc18a2	REVERSE	TCCTGAGGTGGAGGCTGTGAAC	128
