# Supplementary material for: Drinking Water with Saccharin Sodium Alters the Microbiota-Gut-Hypothalamus Axis in Guinea Pig
Source: Animals (Basel). 2021 Jun 23;11(7):1875. doi: 10.3390/ani11071875 (PMC8300211; doi:10.3390/ani11071875)
Supplement: Supplementary file 1 [file animals-11-01875-s001.zip › Supplemental file/Supplementary data 2.pdf]

Supplementary data 2. Quality Control and Tags Statistics of 16srDNA analysis

#Sample_name	Raw_reads(#)	Clean_Reads(#)	Base(nt)	AvgLen(nt)	Q20	GC%	Effective%
CN1	84732	80190	20336709	253	89.31	55.9	94.64
CN2	84637	80146	20333131	253	88.85	55.84	94.69
CN3	85071	80120	20268919	252	90.62	54.21	94.18
CN4	83519	80074	20284098	253	88.03	56.55	95.88
CN5	87949	80086	20310385	253	89.41	55.64	91.06
SS1	85817	80181	20308098	253	86.82	54.89	93.43
SS2	88034	80103	20304908	253	88.1	56.91	90.99
SS3	84936	82865	20407877	253	90.46	53.04	96.23
SS4	85251	80160	20324877	253	87.73	55.84	94.03
SS5	84162	80130	20311169	253	89.34	55.51	95.21

Quality Control 16srDNA analysis

Tags Statistics of 16srDNA analysis

Sample_Name	Total_tag	Taxon_Tag	Unclassified_Tag	Unique_Tag	OTU_num
CN1	80190	72475	0	7715	804
CN2	80146	70713	0	9433	560
CN3	80120	73936	0	6184	522
CN4	80074	60959	0	19115	577
CN5	80086	67281	0	12805	848
SS1	80181	62355	0	17826	518
SS2	80103	69373	0	10730	575
SS3	82865	67801	0	10064	572
SS4	80160	70875	0	9285	555
SS5	80130	68380	0	11750	700
