# Supplementary material for: Drinking Water with Saccharin Sodium Alters the Microbiota-Gut-Hypothalamus Axis in Guinea Pig
Source: Animals (Basel). 2021 Jun 23;11(7):1875. doi: 10.3390/ani11071875 (PMC8300211; doi:10.3390/ani11071875)
Supplement: Supplementary file 1 [file animals-11-01875-s001.zip › Supplemental file/Supplementary data 3.pdf]

Reads statistics of RNA-Seq data

	Reads data statistics		Genome alignment	Align percentage		
sample	raw_reads	clean_reads	total_map	exon	intron	intergenic
CN1	54873662	53803140	48414864(89.99%)	5058467915(69.8194%)	372519984(5.1417%)	1814091862(25.0389%)
CN2	59258102	58107090	52028977(89.54%)	5218206923(67.0146%)	476357362(6.1176%)	2092107076(26.8678%)
CN3	58250506	57408478	52067117(90.70%)	4381560739(69.5873%)	290735013(4.6174%)	1624199560(25.7953%)
SS1	53319052	52260944	47721528(91.31%)	5073585183(71.0503%)	458925737(6.4268%)	1608324452(22.5229%)
SS2	59372024	58196320	53126071(91.29%)	5555690630(69.8794%)	478110423(6.0137%)	1916600169(24.107%)
SS3	65091634	64239206	57584448(89.64%)	5983331110(69.4311%)	418361519(4.8547%)	2215965694(25.7142%)
